# Severe fever with thrombocytopenia syndrome virus infection shapes gut microbiome of the tick vector *Haemaphysalis longicornis*

**DOI:** 10.1186/s13071-024-06204-w

**Published:** 2024-03-05

**Authors:** Yu Sun, Chen Chen, Chenghong Zeng, Qianfeng Xia, Chuanfei Yuan, Hua Pei

**Affiliations:** 1 Key Laboratory of Tropical Translational Medicine of Ministry of Education, International School of Public Health and One Health, Hainan Medical University, Haikou, 571199 Hainan China; 2 Key Laboratory of Tropical Translational Medicine of Ministry of Education, NHC Key Laboratory of Tropical Disease Control, School of Tropical Medicine, The Second Affiliated Hospital, Hainan Medical University, Haikou, 571199 Hainan China

**Keywords:** SFTSV, Gut microbiota, Ticks, *Haemaphysalis longicornis*, *Sphingomonas*

## Abstract

**Background:**

Ticks serve as vectors for a diverse array of pathogens, including viruses responsible for both human and livestock diseases. Symbiotic bacteria hold significant potential for controlling tick-borne disease. However, the alteration of tick gut bacterial community in response to pathogen infection has not been analyzed for any tick-borne viruses. Here, the impact of severe fever with thrombocytopenia syndrome virus (SFTSV) infection on bacterial diversity in the gut of *Haemaphysalis longicornis* is investigated.

**Methods:**

Unfed tick females were artificially infected with SFTSV. The gut samples were collected and the genomic DNA was extracted. We then investigated alterations in gut bacterial composition in response to SFTSV infection through 16S rRNA gene sequencing.

**Results:**

The study found that a reduction in the number of operational taxonomic units (OTUs) in the tick gut following SFTSV infection. However, there were no significant changes in alpha diversity indices upon infection. Four genera, including *Corynebacterium*, *Arthrobacter*, *Sphingomonas*, and *Escherichia*, were identified as biomarkers for the tick gut without SFTSV infection. Notably, the predicted correlation network indicated that the biomarkers *Sphingomonas* and *Escherichia* exhibited positive correlations within the same subcommunity, which was altered upon viral infection.

**Conclusions:**

These findings revealed that the change in tick gut bacterial composition upon SFTSV infection and could facilitate the discovery new target for tick-borne viral disease control.

**Graphical Abstract:**

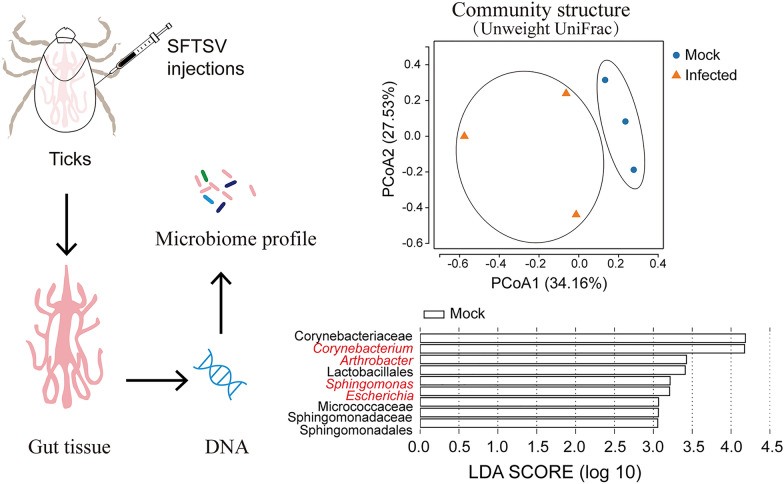

**Supplementary Information:**

The online version contains supplementary material available at 10.1186/s13071-024-06204-w.

## Background

Severe fever with thrombocytopenia syndrome (SFTS) is an emerging human disease that was first identified in central China in 2009 and subsequently found throughout Asia [1, 2]. With a notably high mortality rate of up to 30% and an increasing incidence, SFTS poses a substantial and escalating threat to public health in the Asia region [2]. Currently, no effective therapeutic vaccine or drug is available for SFTS.

SFTS is a tick-borne disease caused by a newly discovered bunyavirus, SFTS virus (SFTSV). SFTSV is maintained in nature through replication cycles in tick vectors and vertebrate hosts. The primary transmitter among these vectors is *Haemaphysalis longicornis* [3, 4], while *Haemaphysalis flava* [5] and *Ixodes sinensis* [6] have also been identified as transmitters of the virus.

Ticks are obligate blood-feeding arthropods known to transmit the widest spectrum of pathogens [7]. In their role as natural vectors, ticks acquire arboviruses either through incidental feeding on virus-infected hosts or cofeeding with infected ticks on a healthy host. Subsequently, the acquired viruses establish infections within the tick, which can then be transmitted to naïve hosts or uninfected ticks during next blood feeding [8]. The tick gut serves as a pivotal natural entry site for arboviruses and represents the primary target for initial virus infection within ticks. The tick gut harbors a diverse microbial community, which can significantly influence the invasion of tick-borne pathogens. In the case of *Ixodes scapularis*, perturbing the gut microbiota could decrease *Borrelia burgdorfer* colonization while increasing susceptibility to *Anaplasma phagocytophilum* infection [9–11].

The tick bacterial community has been observed to be associated with tick-borne pathogens. For instance, in *Rhipicephalus haemaphysaloides*, a higher prevalence of *Babesia microti* among nymphs was observed in conjunction with a diminished density of *Coxiella*-like endosymbiont in larval ticks [12]. A study on *I. scapularis* suggested that *B. burgdorferi*-positive nymphs presented higher levels of *Sphingomonas* [13]. Moreover, following experimental infection of *A. phagocytophilum*, changes in the levels of eight bacteria, including *Acinetobacter* and *Corynebacterium*, were observed within the gut of *I. scapularis* [9].

The research on the changes in the gut microbial community of arthropods in response to viral infection primarily focuses on insects. For example, in the gut of *Aedes japonicus*, *Acinetobacter* showed a decreased relative abundance in response to La Crosse virus infection [14]. Our previous study showed that *Enterococcus* increased in the relative abundance upon baculovirus infection in the gut of *Helicoverpa armigera*, whereas the other bacteria genera decreased [15]. Replenishment of gut *Enterococcus* in germ-free *H. armigera* increased the survival of infected larvae [16]. However, as of current literature, the response of the tick gut microbiome to virus infection has not been reported for any tick-borne viruses.

In our previous study, we employed experimental injection technique to establish SFTSV infection in ticks [17]. In this study, we adopt the approach to establish SFTSV infection in *H. longicornis*. We then investigate alterations in gut bacterial composition in response to SFTSV infection through deep sequencing of tick gut bacterial DNA. Our results provide insights into the change in gut bacterial composition brought about by SFTSV infection. This may offer valuable information for the identification of targets to mitigate viral infection or for the development of biomarkers to detect SFTSV infection in ticks.

## Methods

### SFTSV infection and sample collection

*H. longicornis* were bred in our laboratory as previously described [18]. To establish SFTSV infection, a total of 60 unfed female ticks were selected. Out of these, 30 ticks were injected intrahemocoelically with SFTSV (7.25 × 10^3^FFU), as previously described [17, 19], while the remaining 30 ticks received injections of phosphate-buffered saline (PBS) buffer serving as the control groups. Subsequently, gut samples were collected at 18 days postinfection (dpi) and designated as infected, while the gut samples from the control groups were designated as mock.

### DNA extraction

Gut bacterial DNA was extracted using TRIZol Reagent (Invitrogen™). Briefly, the gut sample was homogenized in TRIZol Reagent, followed by the addition of chloroform and subsequent centrifugation to remove the clear aqueous phase. Ethanol was then introduced to precipitate the DNA, and the resultant pellet was resuspended in 0.1 M sodium citrate in 10% ethanol. After centrifugation, the pellet was washed in 75% ethanol. The DNA pellet was dried for 5 min and then dissolved in 8 mM NaOH, with the pH adjusted to 7 using TE buffer.

### Deep sequencing of gut bacterial DNA

Amplification of the 16S rRNA gene tags (V3–V4) was conducted by polymerase chain reaction (PCR), using 16S rRNA gene-specific primers. Subsequently, the PCR product underwent sequencing on the Illumina Hiseq platform by Huada BGI.

### Bioinformatic analysis

Following the initial sequencing, the obtained reads underwent a stringent filtration process utilizing cutadapt (v.2.6), readfq (v1.0), and iTools Fqtools fqcheck (v.0.25) to generate clean reads. These clean reads were subsequently spliced with FLASH (v1.2.11). The resulting effective tags were then clustered into operational taxonomic units (OTUs) at a 97% identity threshold by employing USEARCH (v7.0.1090). Among the OTUs, the screened sequence with the highest frequency was selected as the representative sequence. Chimeric tags were carefully removed from these representative OTUs using UCHIME (v4.2.40). Finally, the representative OTUs were annotated through RDP classifier (v2.3) with a confidence value of 0.6 based on the Silva 138 database.

Alpha diversity indices, including Shannon, Simpson, Chao, and ace indices, were computed using Mothur (v.1.31.2). Beta diversity was computed using QIIME (v1.80). Afterward, a Principal Coordinate Analysis (PCoA) was conducted using the WGCNA, stats, and ggplot2 packages of the R software.

### Co-occurrence network analysis

Co-occurrence network analysis was performed using the R “ggClusterNet” package and Gephi software (version 0.10.1). For the analysis, only genera that were identified in more than three samples were considered. The ggClusterNet package in R was used for correlation calculation [20]. The correlation value was set as |*r*|> 0.6 and* p* < 0.01. Co-occurrence network pattern analyses were subsequently conducted using Gephi software.

### Data availability

The 16S rRNA gene deep sequencing data used were deposited in the National Center for Biotechnology Information (NCBI) Sequence Read Archive (SRA) under accession number PRJNA1026505.

## Results

### Bacterial 16S rRNA abundance profile

A range of 60,587–73,941 paired-end reads were initially generated (Additional file 1: Table S1). Following the filtering and spicing processes, each gut sample yielded 56,793–64,336 tags. Taxonomic classification based on the Silva 138 database identified 200 OTUs. Notably, the number of OTUs decreased in the tick gut after SFTSV infection.

**Fig. 1:**
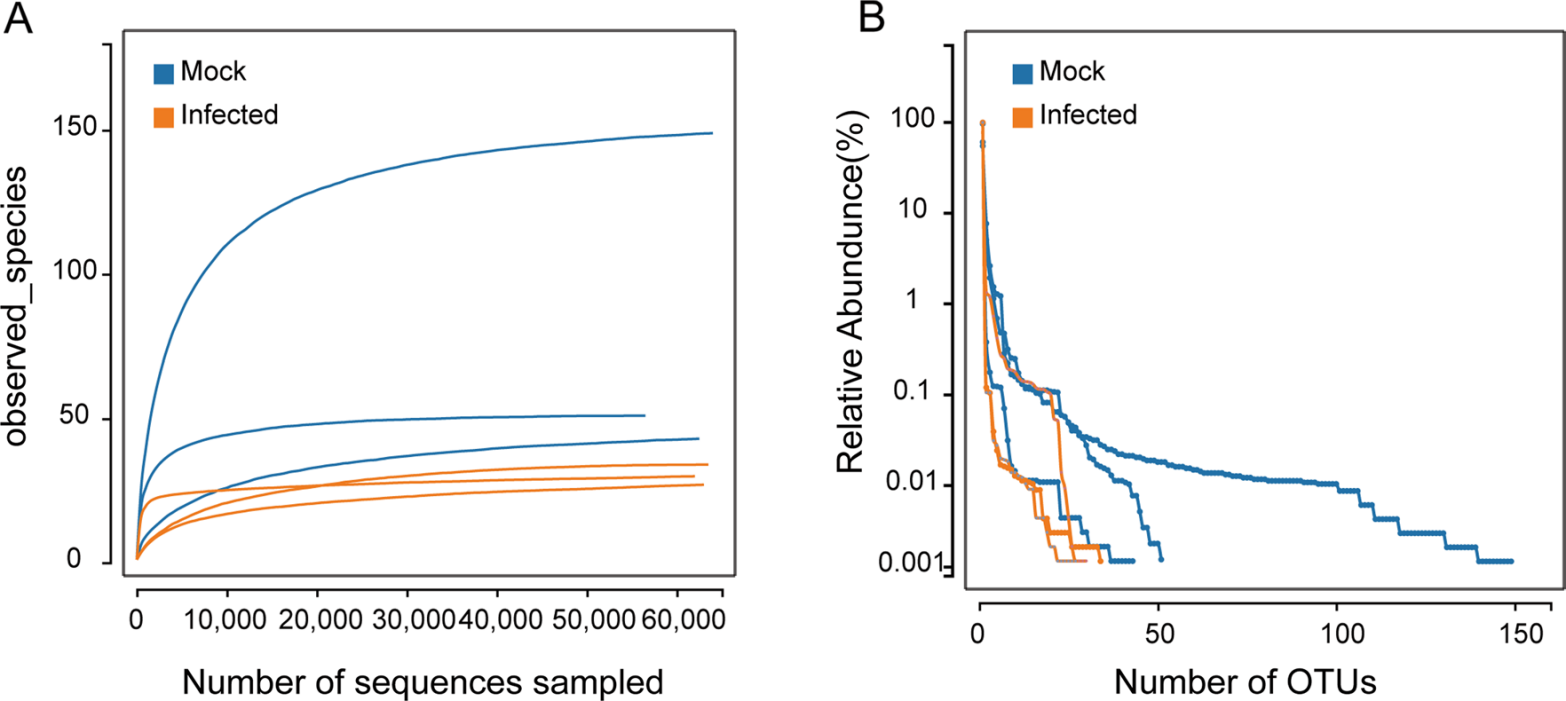
Rarefaction and rank abundance analysis of the gut samples from *Haemaphysalis longicornis*. **A** Rarefaction curves of observed species in the control and infected groups. Mock, gut samples were prepared from unfed females with PBS injection at 18 dpi. Infected, gut samples were prepared from unfed females with severe fever with thrombocytopenia syndrome virus (SFTSV) infection at 18 dpi. **B** Rank abundance diagrams of the gut samples

The rarefaction curve of each sample exhibited saturation, which occurred between 1000 and 10,000 sequences (Fig. 1A), indicating adequate sequencing depth. This observation was corroborated by Good’s coverage (99%). In addition, the rank abundance curves indicated that all gut samples were dominated by *Coxiella* (Fig. 1B)*,* constituting 76.02–99.41% of the population in these samples.

### Gut bacterial composition

**Fig. 2:**
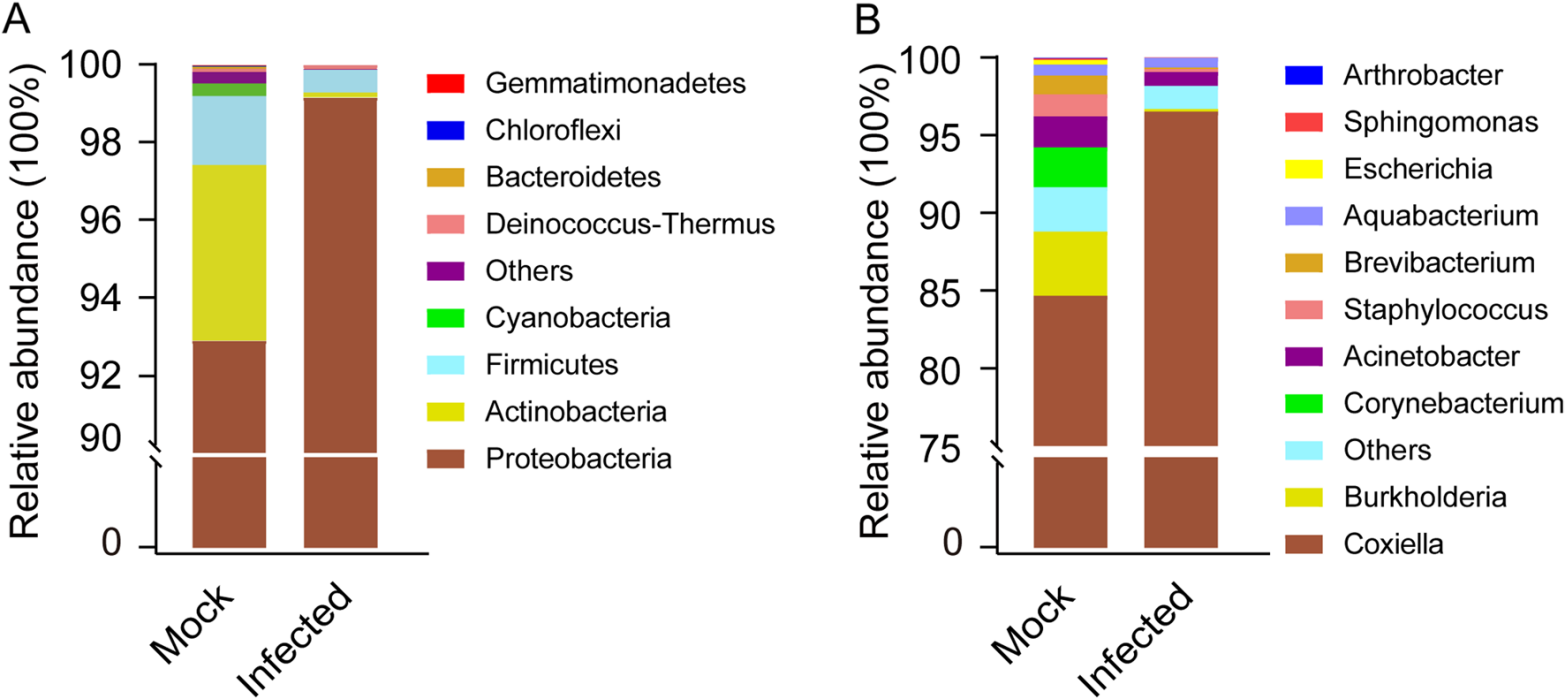
Taxonomic diversity and abundance of each group at phylum (**A**) and genus (**B**) levels

Among the six gut samples, a total of nine phyla, 17 classes, 36 orders, 69 families, and 95 genera were identified. At the phylum level, three phyla—Proteobacteria (93.07%), Actinobacteria (4.35%), and Firmicutes (1.77%)—exhibited a relative abundance greater than 1% in the control group, accounting for 99.14% of the total bacteria (Fig. 2A). In the infected group, these three phyla display relative abundances of 99.14%, 0.12%, and 0.58%, respectively. Interestingly, three trace phyla, namely Cyanobacteria, Chloroflexi, and Gemmatimonadetes, identified in the control group were not identified in the infected group.

At the genus level, six genera with a relative abundance higher than 1% in the control group, namely *Coxiella* (84.68%), *Burkholderia* (4.11%), *Corynebacterium* (2.56%), *Acinetobacter* (2.00%), *Staphylococcus* (1.41%), and *Brevibacterium* (1.21%). These genera collectively accounted for 95.98% of the total bacterial population (Fig. 2B). In the infected group, besides the rise in the relative abundance of *Coxiella* to 96.52%, the remaining five genera decreased below 1%. In addition, the nine genera with a relative abundance of less than 1% were identified in two or three samples in the control group but were not in any samples in the infected group. These genera include *Aliidiomarina*, *Arthrobacter*, *Atopostipes*, *GpI*, *Nesterenkonia*, *Nocardioides*, *Oligella*, *Salinicoccus*, and *Trueperella*.

### Alpha diversity analysis

Alpha diversity was evaluated by Chao, ACE, Shannon, and Simpson indices. Notably, both the Chao and ACE indices exhibited a decrease in the infected gut group, with average values of 83.5 and 83.86, respectively, compared with the mock group, which had average values of 33 and 35.37, respectively (Table 1). The Shannon index also showed a decrease in the infected group (0.22) when compared with the mock group (0.75). However, the Simpson index was upregulated in the infected group (0.93) compared with the mock group (0.73).

**Table 1:** Alpha diversity statistics in each sample from the control and infected groups

Sample	Chao	ACE	Shannon	Simpson
Mock1	46.00	47.17	0.14	0.96
Mock2	153.50	153.10	1.05	0.65
Mock3	51.00	51.32	1.07	0.59
Infected1	33.00	39.83	0.55	0.83
Infected2	32.00	31.86	0.05	0.99
Infected3	34.00	34.41	0.06	0.99

A comparison of the difference in alpha diversity between the control and infected groups showed that the Chao (Wilcoxon test, *W* = 0; *P* = 0.1), ACE (*W* = 0, *P* = 0.1), Shannon (*W* = 1, *P* = 0.2), and Simpson (*W* = 8, *P* = 0.2) indices were all not significantly different. These results collectively suggested that SFTSV infection did not exert a statistically significant impact on the alpha diversity of the gut bacteria.

### Beta diversity analysis

**Fig. 3:**
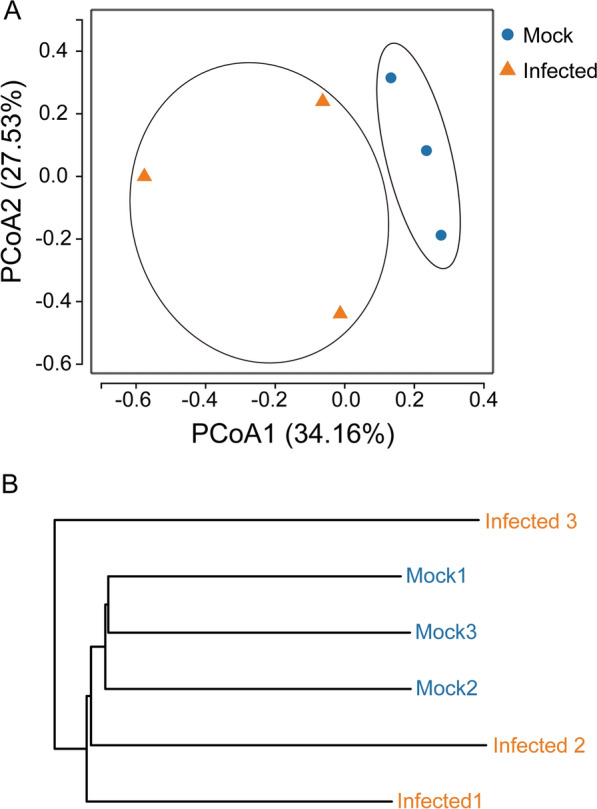
PCoA and UPGMA clustering of the bacterial communities of the gut samples. **A** PCoA plots based on unweighted UniFrac metrics for bacterial communities in the control and infected groups. **B** UPGMA clustering based on unweighted UniFrac Metric for bacterial communities in the control and infected groups

To assess the beta diversity of gut bacterial composition, Principal Component Analysis (PCoA) and unweighted pair group method with arithmetic mean (UPGMA) clustering analysis were employed. PcoA, based on the unweighted UniFrac distances, suggested that the first two principal components, PCoA1 and PCoA2, contribute a large percentage (61.69%) of the total variance of the data, as shown in the two-dimensional scatter plot (Fig. 3A). The results showed that the samples from the infected group clustered separately from those in the control group. Moreover, within the infected group, considerable differences were observed between biological replicates. These findings were consistent with the results obtained from the UPGMA tree analysis based on unweighted UniFrac distances (Fig. 3B). The considerable differences observed between biological replicates within the infected group may be attributed to biological heterogeneity and the complexity of host–microbiome–pathogen interactions.

### SFTSV infection causes changes in taxonomic biomarkers in the gut

**Fig. 4:**
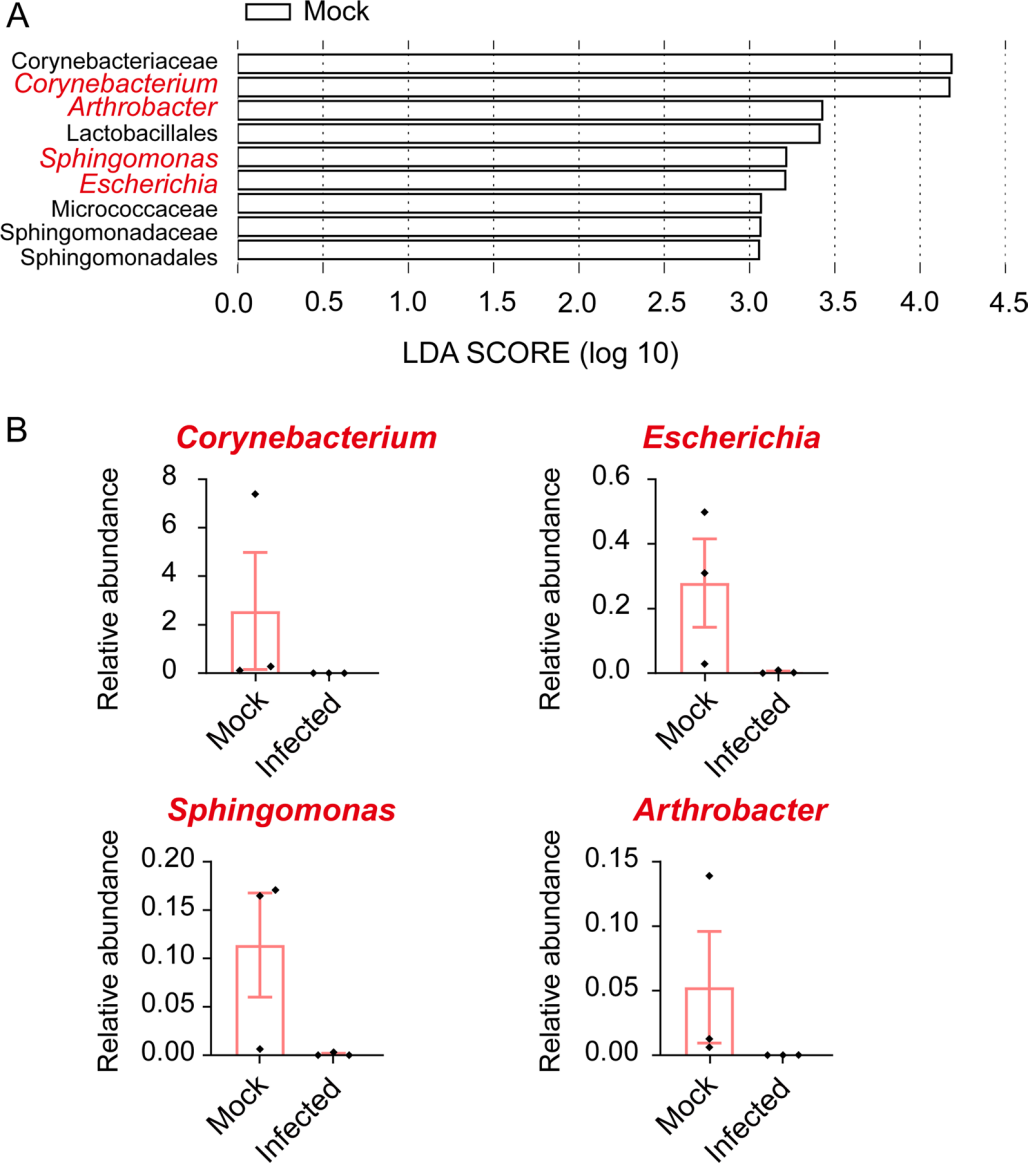
Linear discriminant analysis (LDA) effect size (LEfSe) analysis. **A** Histogram of the LDA scores for different abundant genera in in the control and infected groups (white, enriched in the control group). **B** The relative abundance of enriched genera in the control group

The linear discriminant analysis (LDA) effect size (LEfSe) algorithm approach is widely used approach for identifying biomarkers for high-dimensional data. LEfSe analysis revealed nine bacterial clades showing statistically significant and biologically consistent differences in the control group (Fig. 4A). Among these clades, the two most conspicuously abundant bacterial taxa in the control group were Corynebacteriaceae and *Corynebacterium*. The genus *Corynebacterium* is Gram-positive and belongs to the family Corynebacteriaceae.

Furthermore, the control group exhibited an overrepresentation of genera, including *Arthrobacter*, *Sphingomonas*, and *Escherichia*. Interestingly, all four genera were identified in the three samples of the control group but either were not identified or were present at a low relative abundance in the three samples of the infected group (Fig. 4B). Of note, bacteria belonging to the *Arthrobacter* genus were not identified in any of the three samples from the infected group.

### Alterations in microbiome interactions after SFTSV infection

**Fig. 5:**
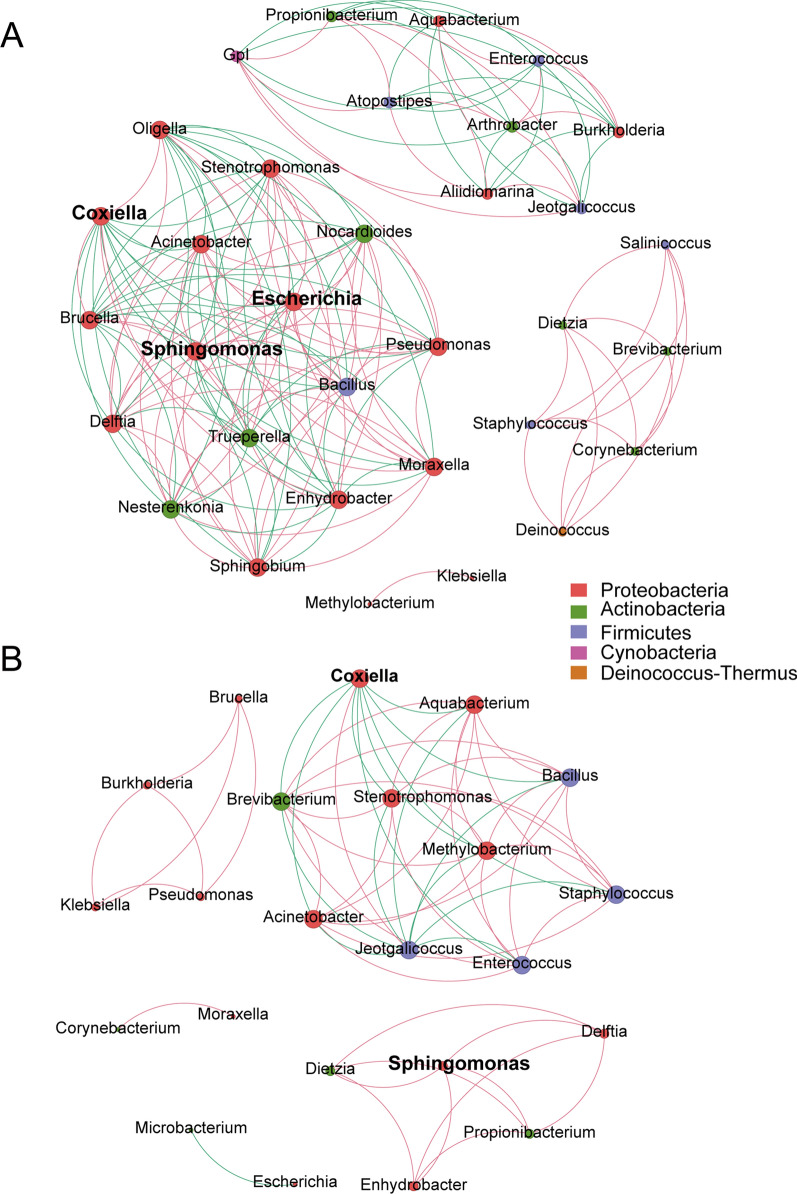
Correlation network analysis. The bacterial microbiome interaction in the control (**A**) and infected (**B**) groups constructed by Gephi. The nodes indicate operational taxonomic units (OTUs) labeled with the bacterial genera. Green edges indicate positive correlation and red edges indicate negative correlation

Building on the concept that microbiomes may operate within niche-specific relationships, we conducted correlation network analysis at the genus level based on the relative abundance of genera. The network of the control group consisted of 33 nodes (genera) and 172 edges (relations) (Fig. 5A), while the network of the infected group revealed 23 nodes (genera) and 63 edges (relations) (Fig. 5B).

Within the control group, the genera were partitioned into four subcommunities, where the biomarkers *Sphingomonas* and *Escherichia* had positive correlations in the same subcommunity (Fig. 5A). In addition, the genus *Coxiella*, which was the most abundant genus in all samples, showed negative interactions with 11 other genera and positive interactions with four other genera.

In the infected group, the genus *Sphingomonas* had positive correlations with four other genera without *Escherichia* (Fig. 5B)*.* In addition, *Enterococcus* showed positive interactions with seven other genera and negative interactions with two other genera in the infected group, while it had positive interactions with three other genera and negative interactions with five other genera in the control group.

## Discussion

While it is crucial to understand the gut microbiome in the context of tick-borne pathogen infection for the development novel strategies in tick-borne disease control, it is important to note that the majority of studies on the tick gut microbiomes have focused on bacterial infections transmitted by ticks. However, the alterations in the tick gut microbiomes following tick-borne viral infection remain poorly understood. Recently, a gut bacterium, whose abundance is regulated by SFTSV infection, can play an important role in modulating viral infection [21]. In our study, we reported that SFTSV infection leads to significant alterations in the gut microbial composition of ticks. Notably, the levels of four bacteria genera, including *Corynebacterium*, *Arthrobacter*, *Sphingomonas*, and *Escherichia*, are reduced in SFTSV-infected female tick guts.

The endosymbiont *Coxiella* plays a vital role in tick development and reproduction, as its reduced abundance has been shown to impair blood intake and decrease reproductive fitness in *H. longicornis* [22, 23]. In addition, *Coxiella* also contributes to larvae-to-nymph transstadial transmission of *B. microti* in *R. haemaphysaloides* [12]. Previous research has indicated that SFTSV infection does not significantly impact tick development and reproductive fitness and larvae-to-nymph transstadial transmission of SFTSV is at high efficiency in *H. longicornis* [24]. Our study showed that *Coxiella* is the dominant bacteria in the gut and SFTSV infection causes no significant change in the gut *Coxiella* in *H. longicornis*. These findings further support the conclusion that SFTSV infection does not affect tick development and reproduction and raise an interesting scientific question about the role of *Coxiella* in larvae-to-nymph transstadial transmission of SFTSV.

*Corynebacterium* is a Gram-positive bacterium that has been identified in the skin of tick-bite patients and associated ticks through transcriptome sequencing [25]. Similarly, *Arthrobacter* is another Gram-positive bacterium, has been identified in field-collected *Haemaphysalis* ticks in our previous study [26]. This study further showed that the two bacteria were identified in the tick gut and their abundance reduced after SFTSV infection. Skin *Corynebacterium* has been shown to promote inflammation in a host with a high-fat diet [27]. *Arthrobacter* also exhibited a decrease in the mosquito gut upon viral infection [14]. Further investigations are needed to clarify the role of these two bacteria in SFTSV infection and to determine whether they are suitable biomarkers for tick gut without SFTSV infection.

*Sphingomonas* is a Gram-negative bacterium and has been identified in the gut of an important agricultural insect pest, *Aphis gossypii*, where it has been associated with mediating imidacloprid resistance [28]. Interestingly, the imidacloprid/flumethrin collar has demonstrated efficacy against tick infestation under field conditions [29]. In addition, *B. burgdorferi*-positive nymphs correlated with higher levels of *Sphingomonas* in *I. scapularis* [13]. Conversely, our study showed that SFTSV-infected ticks presented with lower levels of *Sphingomonas* in *H. longicornis*. These observations raise a hypothesis, the changes in the abundance of *Sphingomonas* in SFTSV-infected ticks may impact tick resistance to imidacloprid/flumethrin collar, that warrants further research.

*Escherichia* is another Gram-negative bacterium that is commonly found with *Sphingomonas* in the gut of *Aedes* mosquito [30]. *Aedes* spp. are the primary vectors of dengue and Zika viruses, which pose significant public health concerns [31, 32]. In this study, the positive interaction between *Sphingomonas* and *Escherichia* in *H. longicornis,*, as well as their decrease following SFTSV infection, suggests that these two genera may function together in virus infection in arthropod vectors. In addition, insect gut *Enterococcus* has been reported to play a supporting role in defending viral infection [16]. Whether *Enterococcus* and the alterations in bacteria interacting with *Enterococcus* within the correlation network after SFTSV infection involve in viral replication in ticks deserves further investigation.

Numerous studies have provided evidences that the gut microbiome can regulate virus infection by modulating insect immunity [15, 33, 34]. For example, the gut bacterium, *Proteus* spp. has been shown to inhibit dengue virus infection by regulating *AMP* gene expression in the gut epithelium of *Aedes aegypti* [35]. In addition, the elimination of gut bacteria resulted in increased resistance against baculovirus and a decrease in the antiviral factor prophenoloxidase [15, 36]. Similar to insects, ticks also possess an immune system to combat against invading pathogens [37–39]. Whether the change in gut microbiome in response to SFTSV infection affects tick immunity, potentially resulting in changes in viral load, is a scientific question that warrants further research.

## Conclusions

Taken together, this study contributes to the body of knowledge concerning the bacterial communities in ticks in response to tick-borne pathogen infections. To the best of our knowledge, this is the first study to characterize the changes that occur in the microbial composition within the tick gut when infected with tick-borne viruses. Our findings establish a baseline understanding of the change in the tick gut in response to virus infection, which can prove helpful in characterizing changes in the tick gut due to infections with other viruses and clarifying the role of candidate bacteria in tick-borne virus infections.

### Supplementary Information

**Additional file 1: Table S1.**Statistics of Tags and operational taxonomic units (OTUs).

## Data Availability

The 16S rRNA gene deep sequencing data used were deposited in the National Center for Biotechnology Information (NCBI) Sequence Read Archive (SRA) under accession number PRJNA1026505.

## Abbreviations

SFTS: Severe fever with thrombocytopenia syndrome
SFTSV: SFTS virus
OUTs: Operational taxonomic units
PCoA: Principal coordinates analysis
