# Supplementary material for: Severe fever with thrombocytopenia syndrome virus infection shapes gut microbiome of the tick vector Haemaphysalis longicornis
Source: Parasit Vectors. 2024 Mar 5;17:107. doi: 10.1186/s13071-024-06204-w (PMC10913621; doi:10.1186/s13071-024-06204-w)
Supplement: Supplementary file 1 — Additional file 1: Table S1.Statistics of Tags and operational taxonomic units (OTUs). [file 13071_2024_6204_MOESM1_ESM.docx]

Table S1. Statistics of Tags and OTUs

| Sample | Raw reads | Clean reads | Tags | OTUs |
| --- | --- | --- | --- | --- |
| Mock1 | 72946 | 69293 | 62892 | 43 |
| Mock2 | 73941 | 69274 | 64336 | 149 |
| Mock3 | 60587 | 57267 | 56793 | 51 |
| Infected1 | 73190 | 69400 | 62356 | 30 |
| Infected2 | 72254 | 68625 | 63036 | 27 |
| Infected3 | 71966 | 68686 | 63546 | 34 |
